# A human-derived neurovascular unit *in vitro* model to study the effects of cellular cross-talk and soluble factors on barrier integrity

**DOI:** 10.3389/fncel.2022.1065193

**Published:** 2022-12-01

**Authors:** Chiara Barberio, Aimee Withers, Yash Mishra, Pierre-Olivier Couraud, Ignacio A. Romero, Babette Weksler, Róisín M. Owens

**Affiliations:** ^1^Department of Chemical Engineering and Biotechnology, University of Cambridge, Cambridge, United Kingdom; ^2^Institut Cochin, Centre National de la Recherche Scientifique UMR 8104, Institut National de la Santé et de la Recherche Médicale (INSERM) U567, Université René Descartes, Paris, France; ^3^Department of Biological Sciences, The Open University, Milton Keynes, United Kingdom; ^4^Department of Medicine, Weill Medical College of Cornell University, New York, NY, United States

**Keywords:** neurovascular unit (NVU) model, blood-brain barrier (BBB), Transwell, *in vitro*, human immortalized cell lines

## Abstract

The blood-brain barrier (BBB) restricts paracellular and transcellular diffusion of compounds and is part of a dynamic multicellular structure known as the “neurovascular unit” (NVU), which strictly regulates the brain homeostasis and microenvironment. Several neuropathological conditions (e.g., Parkinson’s disease and Alzheimer’s disease), are associated with BBB impairment yet the exact underlying pathophysiological mechanisms remain unclear. In total, 90% of drugs that pass animal testing fail human clinical trials, in part due to inter-species discrepancies. Thus, *in vitro* human-based models of the NVU are essential to better understand BBB mechanisms; connecting its dysfunction to neuropathological conditions for more effective and improved therapeutic treatments. Herein, we developed a biomimetic tri-culture NVU *in vitro* model consisting of 3 human-derived cell lines: human cerebral micro-vascular endothelial cells (hCMEC/D3), human 1321N1 (astrocyte) cells, and human SH-SY5Y neuroblastoma cells. The cells were grown in Transwell hanging inserts in a variety of configurations and the optimal setup was found to be the comprehensive tri-culture model, where endothelial cells express typical markers of the BBB and contribute to enhancing neural cell viability and neurite outgrowth. The tri-culture configuration was found to exhibit the highest transendothelial electrical resistance (TEER), suggesting that the cross-talk between astrocytes and neurons provides an important contribution to barrier integrity. Lastly, the model was validated upon exposure to several soluble factors [e.g., Lipopolysaccharides (LPS), sodium butyrate (NaB), and retinoic acid (RA)] known to affect BBB permeability and integrity. This *in vitro* biological model can be considered as a highly biomimetic recapitulation of the human NVU aiming to unravel brain pathophysiology mechanisms as well as improve testing and delivery of therapeutics.

## Introduction

Brain homeostasis and neuronal function are maintained by the blood-brain barrier (BBB), a highly selective impermeable cell monolayer which separates the bloodstream from the brain tissue and regulates the diffusion of molecules and ions between the two. The BBB contributes to the prevention of pathogens and cells of the immune system from accessing the brain parenchyma and triggering neuroinflammation (Knox et al., 2022). This is due to the fact that brain endothelial cells express specific junctions and transporters, thus making the BBB a physical and metabolic barrier (Oldendorf, 1971). The transport of water-soluble molecules needed by the brain is enabled by influx transporters, whilst waste and neurotoxic agents are actively pumped out by efflux transporters (Oldendorf, 1971; Abbott et al., 2010). Amongst others, the drug-transporter, P-Glycoprotein (P-Gp), rejects most large drugs (>400 Da) and makes drug delivery into the brain very challenging (van der Helm et al., 2016). Several neuropathological conditions, including stroke, epilepsy, Parkinson’s and Alzheimer’s disease, are associated with BBB dysfunction (Cai et al., 2018; Modarres et al., 2018). BBB disruption is a pathophysiological feature of 2 of the top 10 causes of death worldwide, stroke and dementia; however, most of the underlying mechanisms still remain unclear (Abdullahi et al., 2018; Cai et al., 2018; Jiang et al., 2018; World Health Organization [WHO], 2020).

The BBB is part of a highly specialized complex three-dimensional (3D) structure composed of pericytes, glial cells, and neurons in addition to endothelial cells (Cecchelli et al., 2007), known as the “neurovascular unit” (NVU) (Zenaro et al., 2017). Human brain endothelial cells are characterized by specific endothelial markers like vascular endothelial cadherin (VE-Cadherin) and tight junction proteins (TJs), such as zona occludin-1 (ZO-1), occludin and claudin-5 which restrict paracellular permeation (Rahman et al., 2016). Pericytes wrap around the endothelial cells and contribute to various brain functions, including BBB development and maintenance, brain inflammation and control of cerebral blood flow (Ito et al., 2019). Glial cells include astrocytes, microglia, and oligodendroglia (Yu et al., 2020), which play an essential role in neuronal survival and growth, neuroprotection and myelin formation, respectively. The most abundant glial cells are astrocytes, which uniquely express glial fibrillary acidic protein (GFAP) and are typically stellate cells with processes (Vasile et al., 2017). Besides promoting neuronal survival and contributing to synapse formation, synaptosome engulfment, and neurotransmitter transmission, astrocytes participate in brain homeostasis through BBB formation and function and end-feet that contact blood vessels (Cecchelli et al., 2007; Vasile et al., 2017). Neurons help control our bodies by sending signals to other cells through their long, branching dendrites (Daneman and Prat, 2015). They express specific structural, mature neuronal markers such as β III-tubulin, microtubule-associated protein-2 (MAP2), and synaptic genes (Kovalevich and Langford, 2013).

Due to toxicity or low efficacy, over 90% of drugs that pass animal testing fail to pass human clinical trials (Van Norman, 2019). This may in part be due to inter-species variability as several human-specific proteins and protein isoforms pertinent to development and disease physiopathology are not expressed by animal models (Garner, 2014; Akhtar, 2015; Van Norman, 2019). It is crucial to develop complex, highly biomimetic, human-based models of the NVU to better understand BBB development, function and the mechanisms connecting BBB dysfunction to neuropathological conditions, and thus design better drug delivery strategies as well as effective new treatments, whilst reducing time and resource requirements.

Co-culturing brain endothelial cells with the other types of NVU cells has been shown to improve BBB integrity in several studies (Gaillard et al., 2001; Boveri et al., 2005; Cucullo et al., 2008; Malina et al., 2009; Hatherell et al., 2011; Lippmann et al., 2014; Appelt-Menzel et al., 2017). This mainly occurs through the cellular cross-talk *via* secretion of soluble factors into the media (e.g., astrocyte-conditioned media enhances BBB tightness) as well as *via* direct cell-cell interaction (e.g., astrocyte end-foot-endothelial cells contact) (Malina et al., 2009; Hatherell et al., 2011). In the case of the immortalized cell line, human cerebral microvascular endothelial cells (hCMEC/D3) (Weksler et al., 2013), co-cultured with astrocytes increased transendothelial electrical resistance (TEER) by about 1.5-fold after 5 days (Hatherell et al., 2011). Using non-human cells for modeling is cost-effective and easier to obtain, yet they exhibit intrinsic differences in functions and phenotype. Other more sophisticated tissue-engineered approaches, such as spheroids and microfluidic-based models offer a closer recapitulation of the *in vivo* microenvironment but they are expensive and complicated to reproduce (Stone et al., 2019). By contrast, human immortalized cells provide a comparably accessible and easy to use option for *in vitro* cell-based models, as representative of human biology for translational research (Geraghty et al., 2014; Barbosa et al., 2015; McKee and Chaudhry, 2017; Lipps et al., 2018).

**FIGURE 1:**
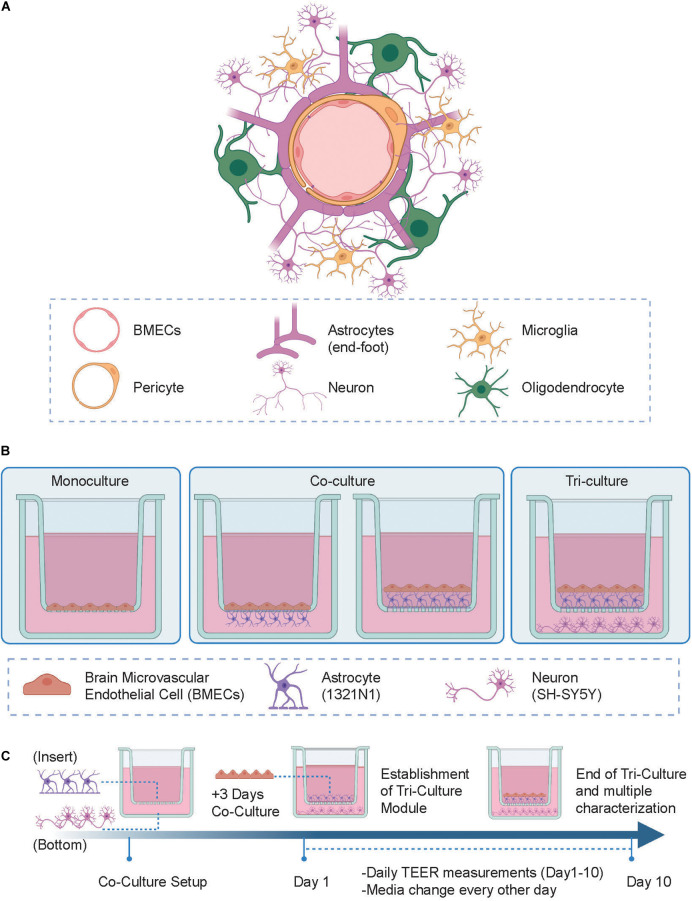
**(A)** Schematic representation of the human neurovascular unit (NVU) *in vivo*. **(B)** Experimental setup of the tri-culture NVU model showing the various configurations of cells tested. (Left) In the monoculture setup brain microvascular endothelial cell (BMECs) are seeded in the apical side of the insert; (Center) in the co-culture setup, BMECs and astrocytes are either separated by the insert porous membrane where astrocytes are seeded on the underside of the membrane (i.e., “indirect” contact co-culture) or both together in direct contact in the apical side of the insert (i.e., “contact” co-culture); (Right) in the tri-culture model, neurons are added to the co-culture setup and seeded on the bottom of a 24-wellplate. **(C)** Development of the *in vitro* NVU model protocol, where astrocytes and neuronal cells are both seeded on day 0 (this step is skipped in the monoculture model protocol) followed by BMECs cells seeding on day 3. Daily transendothelial electrical resistance (TEER) measurements were then taken until day 10 after which the system was further characterized using multiple other analytical techniques. Figure created with www.biorender.com.

There is a compelling need to develop human-based *in vitro* models of the NVU comprehensive of multiple cell types as a robust tool to investigate their interactions and their influence on barrier integrity. Transwell systems are simple, commonly used, semi-permeable supports for creating a variety of *in vitro* BBB models with different configurations (Figure 1). These inserts represent a robust approach to control cell culture in real-time and non-invasively, enabling the study of barrier formation and integrity (Allen and Bayraktutan, 2009; Cecchelli et al., 2014; Simöes Da Gama and Morin-Brureau, 2022). The membrane creates 2 compartments, where endothelial cells can be grown as a monolayer in the upper “apical” chamber representing the “blood” side, whilst the lower “basolateral” chamber represents the “brain” compartment (Helms et al., 2015; Stone et al., 2019). Furthermore, this model compartmentalization enables separate access to both apical and basal sides, thus allowing dedicated studies for drug treatment or cell-type specific visualization over the time course of the experiment.

In this study we developed a human-based triculture model of the NVU using Transwell systems as supports for generating a variety of configurations with extra-layers of complexity (i.e., monoculture, co-culture, and tri-culture). After establishing the multiple cell culture seeding protocol and defining the optimal cell culture conditions, these NVU setups underwent daily barrier integrity evaluation (i.e., TEER measurements) and multiple end-point characterization where properties like barrier permeability, biomarker expression, neural cell viability, and outgrowth were evaluated and compared between conditions. We observed that at day 10 of cell culture the highest TEER value was reached in the tri-culture setup, suggesting that the co-presence of astrocytes and neurons in the model contributes to barrier formation and tightness over time. Expression of biomarkers specific to each NVU cell type was confirmed *via* immunofluorescence and was found to be consistent across the different model configurations. Finally, to validate the model we tested soluble factors well-known for either improving [i.e., retinoic acid (RA)] or perturbing [i.e., Lipopolysaccharides (LPS)] barrier strength, showing the specific effects on the TEER values upon incubation.

We believe this *in vitro* biological model can be considered as a highly biomimetic recapitulation of the human NVU that can be used as a versatile tool for translational research to investigate underlying factors associated with neuropathological disorders and for drug delivery optimization.

## Materials and methods

### Cell culture

Human cerebral micro-vascular endothelial cells (hCMEC/D3) were a gift from Dr. Pierre-Olivier Couraud of the Institut Cochin, INSERM, Paris, France (Weksler et al., 2013). The hCMEC/D3 used for the experiments were between passage 32 and 34. T-flasks were coated with rat-tail collagen type I solution (Thermo Fisher, United Kingdom) at a concentration of 0.1 mg/ml in DPBS (Sigma Aldrich, United Kingdom) and were incubated for 1 h at room temperature. hCMEC/D3 were sub-cultured in Endothelial Basal Medium-2 (EBM-2; Lonza Group Ltd., United Kingdom) supplemented with 0.025%v/v human epidermal growth factor (rhEGF), vascular endothelial growth factor (VEGF), and insulin-growth factor-1 (IGF), 0.1%v/v human fibroblast growth factor (rhFGF), 0.1%v/v gentamycin and ascorbic acid, 0.04%v/v hydrocortisone, and 2.5%v/v Fetal Bovine Serum (FBS) (all supplied by Lonza). Cell culture medium was changed every 2 days. Human 1321N1 astrocytoma cells were purchased from the European Collection of Authenticated Cell Cultures and were below passage 8 for all the experiments. They were grown in High glucose DMEM (Sigma-Aldrich) supplemented with 10%v/v FBS (Life Technologies–Invitrogen), 1%v/v Glutamax (Life Technologies, United Kingdom), and 1%v/v penicillin/streptomycin (Invitrogen, United Kingdom), which will be referred to as “1321N1 media.” Human SH-SY5Y neuroblastoma cells (European Collection of Cell Cultures, ECACC, Sigma-Aldrich) below passage 10 were maintained in 1:1 Minimum Essential Media (MEM; Sigma Aldrich, United Kingdom) and Nutrient Mixture F-12 Ham (Sigma-Aldrich) with 15% FBS (Life Technologies–Invitrogen, USA) and 1% Non-Essential Amino Acids (NEAA 100X, Sigma Aldrich, United Kingdom) and 1% Glutamax 1–100X (Life Technologies, United Kingdom) and 1% penicillin/streptomycin (Invitrogen, United Kingdom). All cells were cultured in a humidified incubator at 37°C and 5% CO_2_. The co-culture and tri-culture media was made with EBM-2 (Lonza Group Ltd., United Kingdom) supplemented with 5% FBS (Life Technologies–Invitrogen, USA), 0.1% v/v rhFGF, 1.4 μM hydrocortisone, 5 μg/ml ascorbic acid (all supplied by Lonza), 0.1% v/v chemically modified lipid concentrate (CDLC, Thermo Fisher, United Kingdom), 2.5% v/v HEPES solution (Thermo Fisher, United Kingdom), and 1% penicillin/streptomycin (Invitrogen, United Kingdom). All media was filtered through a 0.2 μm filter before use.

For experiments, cells were cultured in the configurations shown in Figure 1A on 0.4 μm pore Greiner Bio-One ThinCert™ hanging cell culture inserts, which have a 0.33 cm^2^ PET membrane, in 24-well plates. Once hCMEC/D3 cells were seeded, all cells were grown in EBM-2 medium (Lonza) supplemented with 10 mM HEPES, 1 ng/ml basic human FGF (rhFGF), 1.4 μM hydrocortisone, 5 μg/ml ascorbic acid, penicillin–streptomycin, chemically defined lipid concentrate, and 5% FBS to facilitate co-cultures and TJ formation. For the monoculture conditions, hCMEC/D3 cells were seeded on top of the insert membranes at 100,000 cells/cm^2^. For the co-culture conditions, 1321N1 (astrocyte) cells were seeded on either the top or bottom side of the membrane of each insert at 40,000 cells/cm^2^ on the 1st day and then hCMEC/D3 cells were seeded on top of each membrane at 100,000 cells/cm^2^ after 3 days. For the tri-culture conditions, 1321N1 (astrocyte) cells were seeded on the top side of the membrane of each insert at 40,000 cells/cm^2^ and 10,000 SH-SY5Y cells were seeded on the bottom of each corresponding well on the 1st day and then hCMEC/D3 cells were seeded on top of each membrane at 100,000 cells/cm^2^ after 3 days. The cells were cultured for 10 days after the seeding of hCMEC/D3 cells with the media changed every other day, and then further characterization was carried out. The tri-culture model was then set up in the same way described above and validated 14 days after the seeding of hCMEC/D3 by studying the effects of the addition of LPS from *Escherichia coli* O111:B4 (Sigma-Aldrich) at 100 ng/ml, sodium butyrate (NaB; Sigma-Aldrich) at 100 μM, or RA (Sigma-Aldrich) at 10 μM to the cell culture media for 24 h using an EVOM Volt-Ohm meter.

### Transendothelial electrical resistance measurements

For each well, TEER was measured every day using an EVOM^®^Volt-Ohm meter [World Precision Instruments (WPI), Sarasota, FL, USA] endowed with STX electrodes. The measured values were blanked using read-outs from hanging cell culture inserts in media, but with no cells. During measurements, the longer electrode was kept outside the Transwell insert within the well and the shorter one inside for probing the BCMECs monolayer. Readings were repeated trice to provide reproducible measurements. Cell-free collagen pre-coated inserts were also measured and used as control in the TEER baseline calculations. On day 15 of cell culture, TEER was measured upon 24 h incubation with 100 ng/ml LPS, 100 μM NaB and 10 μM RA.

### Lucifer yellow permeability assay

Paracellular permeability was evaluated using the fluorescent marker, Lucifer yellow (LY, Lucifer yellow CH, lithium salt, Thermo Scientific). A transport buffer composed of 1%v/v HEPES solution added to Hank’s Balanced Salt Solution (HBSS) (1×, Gibco) was added to wells of a 24-well plate and placed in a humidified incubator at 37°C, 5% CO2. The hanging cell culture inserts were then rinsed with the transport buffer and then placed in the 24-well plate with the transport buffer. LY solution (300 μM) was then added to the apical side of the inserts (0.3 ml per insert) and the plate was incubated at 37°C and 5% CO_2_ for 1.30 h. Samples taken from both the apical and basolateral sides of each insert were then analyzed using a Tecan Spark multimode microplate reader to record fluorescence values (excitation/emission wavelengths at 428/536 nm). A standard curve was then used to estimate the LY concentration of each sample and then apparent permeability (Papp, cm/s) was calculated using:


$$Papp=\frac{\left[\frac{\text{Flux}\times {\text{Vol}}_{\text{B}}}{\text{T}}\right]}{\left[\frac{1}{\text{A}\times \left[\text{C}\right]}\right]}whereFlux=\frac{{\text{LY}}_{\text{B}}\times {\text{Vol}}_{\text{B}}}{{\text{LY}}_{\text{B}}\times {\text{Vol}}_{\text{A}}}\times 100$$

LY_A_ and LY_B_ are the concentrations of LY in the apical and basal compartments, respectively. Vol_A_ and Vol_B_ are the volumes in the apical and basal sides, respectively. T (seconds) is the time of incubation. C is the initial concentration of LY on the apical side (300 μM) and A is the area of the membrane.

### Immunofluorescence staining

Cells were washed with Dulbecco’s–Phosphate Buffered Saline (PBS) having Calcium and Magnesium (Sigma-Aldrich) at the beginning and in between steps. They were fixed with 4% paraformaldehyde by incubating at room temperature for 5 min. The permeabilization was done through incubation in 0.25% Triton in PBS for 10 min at room temperature and the blocking step was carried out by incubating cells in 1% BSA in PBST (0.05% Tween 20 in PBS) for 30 min at room temperature. A 1:50 rabbit polyclonal anti-ZO 1 (617300, Invitrogen, United Kingdom), 1:400 rabbit monoclonal VE-Cadherin (D87F2, Cell Signalling Technology, United States), and 1:400 rabbit polyclonal anti-glucose transporter GLUT1 (ab15309, Abcam, United Kingdom), were used as primary antibodies in 1% BSA in PBS and added to the samples, which were then incubated overnight at 4°C. Cells were then incubated for 1 h at room temperature with 1:500 of Alexa Fluor 488 Goat anti-Rabbit IgG (Invitrogen, United Kingdom) in 1% BSA in PBS. Lastly, the samples were incubated for 5 min at room temperature with 1 μg/ml Hoechst 33 342 (Sigma Aldrich, United Kingdom), mounted, and examined with a confocal microscope (ZEISS LSM 800).

### Neurite outgrowth staining and tracing

A Molecular Probes™ Neurite Outgrowth Staining Kit (Life Technologies, United Kingdom) was used according to the manufacturer’s protocol for measuring the neuron viability and neurite outgrowth of SH-SY5Y cells in both co-culture and tri-culture models. Briefly, 10 μl of Cell Viability Indicator and 10 μl of Cell Membrane Stain were added in 10 ml of PBS containing calcium and magnesium (1× working Stain Solution). After incubation for 20 min at 37°C and 5% CO_2,_ cells were washed once with PBS and 1× working Background Suppression Dye (10 μl per 1 ml PBS) was added to the wells for confocal imaging (ZEISS LSM 800) and fluorescence quantification using a Tecan Spark microplate reader. For confocal microscopy, the Cell Viability Indicator dye settings were: excitation 495 nm and emission 515 nm; for Cell Membrane Stain: excitation 555 nm and emission 565 nm. Plate reader settings: Cell Viability Indicator: excitation 483 nm and 525 nm emission, bandwidth 10 nm; Cell Membrane Stain: excitation 554 nm and 567 nm emission, bandwidth 5 nm. Quantitative readouts were obtained from three replicates for each condition except for the BMECs-neurons dataset where only 2 datapoints were generated out of 3 (“over” readout). Neurite outgrowth analysis was performed using the semi-automated ImageJ plugin, NeuronJ. For each condition (i.e., BMECs-neurons, astrocytes-neurons, and tri-culture), three confocal images were used for analysis and 90 neurites were manually traced for quantification.

### Statistical analysis

Analysis of statistical differences was performed, specifically: One-way ANOVA analysis was used for TEER analysis and comparison. Dunnett’s multiple comparison test was used for the TEER values comparison between the control tri-culture model (untreated) vs. the treated samples; Tukey’s multiple comparison test was used to compare % TEER change between each condition. Statistical analysis was performed with GraphPad Prism9.0.0 (121). Data were represented as mean of three replicates ± standard deviation.

## Results and discussion

### Development of the *in vitro* neurovascular unit model protocol

To generate a biomimetic model of the human NVU, the following human immortalized cell lines were cultured: hCMEC/D3 cells as representative of brain microvascular endothelial cell (BMECs), 1321N1 cells for astrocytes and SH-SY5Y cells for neurons. The schematics of the NVU *in vivo* architecture and *in vitro* models developed in our study are shown in Figures 1A,B. Using Transwell systems, we developed 3 different model setups with increasing level of complexity and biomimicry, namely: (1) a monoculture model (i.e., BBB), the most reductionist configuration where BMECs were located in the apical side of the insert (Figure 1B, left); (2) a co-culture setup in which BMECs were seeded on the apical side along with 1321N1 seeded either on the underside of the insert (i.e., “indirect” contact co-culture, Figure 1B, center-left) or BMECs seeded on top of 1321N1 cells on the apical side of the insert (i.e., contact co-culture, Figure 1B, center-right). The establishment of these two co-culture conditions was aimed to test changes in barrier formation and its properties depending on the type of physical contact between BMECs and astrocytes (i.e., intimate and direct cell-cell interaction in the contact co-culture); lastly, (3) a tri-culture setup with both BMECs and 1321N1 growing in contact on the inside of the insert and SH-SY5Y cells seeded on the bottom of the well plate (Figure 1B, right). Although the tri-culture model is lacking other important NVU cell types (e.g., pericytes, microglia, oligodendrocytes), it comprises the main components of a functional NVU, such as the endothelial barrier representative of the brain vasculature, neural cells for emulating the brain tissue and glial cells acting as mediators between the two modules. Thus, the tri-culture best recapitulates the physiological and structural composition of the human NVU compared to the monoculture and co-culture layouts. Indeed, it has been previously reported that the crosstalk and exchange of growth factors between each cell type are enhanced and promoted in such multicellular microenvironment than in more simplified and reductionist single cell type models (Schreiner et al., 2022). Following optimization of the cell culture media composition, cell density, and cell seeding strategies we established a 10-day cell culture protocol where each cell type of the model was seeded in the respective compartment at a specific time point of the study (Figure 1C).

### Monitoring barrier formation and integrity

**FIGURE 2:**
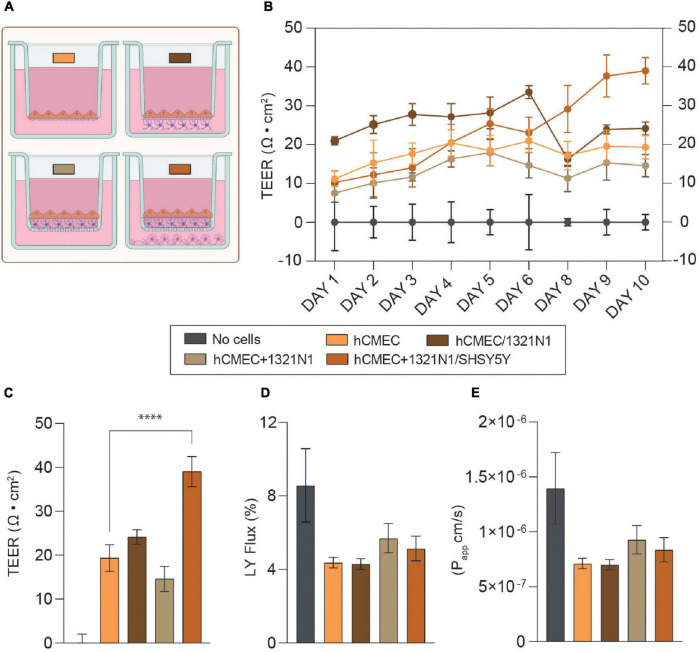
**(A)** Schematics of the neurovascular unit (NVU) setups used for EVOM measurements and permeability assay. The color code refers to the legend in this figure. Created with Biorender.com. **(B)** Mean transendothelial electrical resistance (TEER) values (Ω cm^2^) for hCMEC in monoculture, hCMEC, and 1321N1 co-culture (hCMEC/1321N1 as “indirect” contact and hCMEC+1321N1 as “contact” co-culture) and hCMEC+1321N1/SHSY5Y tri-culture. Measurements were performed daily for 10 days from Transwell inserts in 24-wellplates (pore size 0.4 μm, area 0.33 cm^2^). **(C)** Bar chart representing TEER values at day 10 post-seeding in all NVU model setups. The difference between monoculture, co- and tri-culture was assessed using a one-way ANOVA with ^****^p < 0.0001. The error bars represent the standard deviation of at least 3 replicates for each condition (mean ± SD). **(D,E)** Lucifer yellow (LY) flux and apparent permeability (Papp) bar charts for monoculture, co-culture, and tri-culture, showing low paracellular permeability to LY (<1 × 10^– 6^ cm/s) in all the conditions in the range of values observed elsewhere. The error bars represent the standard deviation of at least 3 replicates for each condition (mean ± SD).

Transendothelial electrical resistance (TEER) measurement is an extensively used parameter to quantitatively and non-invasively evaluate barrier formation and stability in *in vitro* barrier model systems (i.e., endothelium or epithelium monolayers) (Srinivasan et al., 2015). In our study, TEER measurements were performed daily in all NVU model configurations 24 h post-endothelial cell seeding (i.e., Day 1, Figure 1C). As reported in Figure 2A and Supplementary Figure 1, each NVU model configuration is characterized by different initial TEER values which undergo fluctuations over the cell culture timeline. However, from day 8 of cell culture a significant increase in TEER was observed in the tri-culture setup, reaching plateau at day 10 (∼ 40 Ω cm^2^, ^****^p < 0.001). Lower TEER values were found in the other model configurations (i.e., monoculture ∼20 Ω cm^2^, “indirect” contact co-culture >20 Ω cm^2^, “contact” co-culture >10 Ω cm^2^), Figure 2B. These findings are in line with previous studies showing similar, increased TEER values when endothelial cells are co-cultured with other cell types of the NVU (Ito et al., 2019; Stone et al., 2019; Gericke et al., 2020). It is worth mentioning that the BBB *in vivo* has been estimated to reach higher TEER values which are difficult to reproduce *in vitro* (>1000 Ω⋅cm^2^) (Sivandzade and Cucullo, 2018; Bhalerao et al., 2020; Erickson et al., 2020); in addition, most of the *in vitro* BBB model developed to date exhibit different ranges of TEER values depending on the cell source in use [i.e., iPSCs, primary cells or rodent-derived cells, (Srinivasan et al., 2015)], the cell culture conditions and timelines. Following previous studies (Elbakary and Badhan, 2020), we also tested the effects of shear stress on the monoculture barrier tightness with respect to the static condition (Supplementary Figure 2), noting that induced shear stress has beneficial influence on barrier formation and tightness in hCMEC/D3 derived BBB. As a simplified means of applying shear stress, we used the orbital rotation approach by setting the rotator to 150 rpm (i.e., ∼7–8 dyn/cm^2^ of shear) as described in previous studies (Barichello, 2019). However, the remaining cell types of the model (i.e., 1321N1 and SHSY5Y cells) did not exhibit a healthy phenotype when subjected to orbital shaking, which is likely explained by the fact that within the NVU microenvironment the bloodstream is only interfacing with endothelial cells of the BBB, whereas astrocytes and neurons are not exposed to its physical cues. Thus, the application of flow induced shear stress was not carried forward to the optimized model.

Taking into consideration the reductionist approach of a tri-culture model of the NVU, we demonstrated that the co-presence of astrocytes and neurons within the biological model promotes barrier formation and enhances its integrity with respect to the barrier monolayer (Figures 2A,B).

Next, permeability assays were carried out for each biological model at day 10 of the protocol, as an endpoint following the final TEER recordings. In the case of the tri-culture setup, this timepoint was considered optimal for testing the barrier permeability given the higher TEER value (∼40 Ω cm^2^) should have corresponding low permeability values. It is well-known that BBB endothelial cells exhibit very limited paracellular permeability to hydrophilic molecules, due to the formation of TJs. BBB permeability was assessed by measuring the flux of a small fluorescent marker across the barrier generated, known as LY (MW = 457 Da). Both the LY flux (%) and the apparent permeability (Papp) were calculated in each condition for comparison. Although there is no statistical significant difference in barrier permeability between the monoculture and the other model configurations, we observed lower permeability values (<1 × 10^–6^ cm/s) in all the setups with respect to the cell-free control and similar to those reported in most of the studies using hCMEC/D3 as barrier model (Figures 2D,E; Poller et al., 2008; Eigenmann et al., 2013; Zhao et al., 2019). Interestingly, a decrease in Papp was also found when both the monoculture (i.e., ≤5 × 10^–7^ cm/s dynamic vs. >6 × 10^–7^ cm/s static) and tri-culture (i.e., ∼7.1 × 10^–7^ cm/s dynamic vs. ∼8.4 × 10^–7^ cm/s static) were exposed to shear stress (Supplementary Figure 3), supporting the theory of physical cues like shear stress as enhancers of barrier strength and tightness.

It is worth noting that the use of different cell lines, cell density, Transwell membrane coating, membrane porosity and cell culture media composition are all factors contributing to different TEER and paracellular permeability values across NVU *in vitro* models generated in similar studies (Ito et al., 2019; Stone et al., 2019; Bhalerao et al., 2020; Gericke et al., 2020; Simöes Da Gama and Morin-Brureau, 2022). From day 5 of cell culture we observed an overgrowth of astrocytes in both the co-culture and tri-culture setups, meaning that the endothelial cells sitting on top of the astrocytes could be prevented from forming an even and flat barrier monolayer, resulting in a wavy and groovy cellular monolayer. Although the TEER values seem to not be affected by such a phenomenon, this could explain the slightly higher apparent permeability found in these multicellular model configurations with respect to the monoculture where cells adhere directly on the insert porous membrane (Figures 2C–E).

### Identification of neurovascular unit-relevant cell types and barrier visualization

**FIGURE 3:**
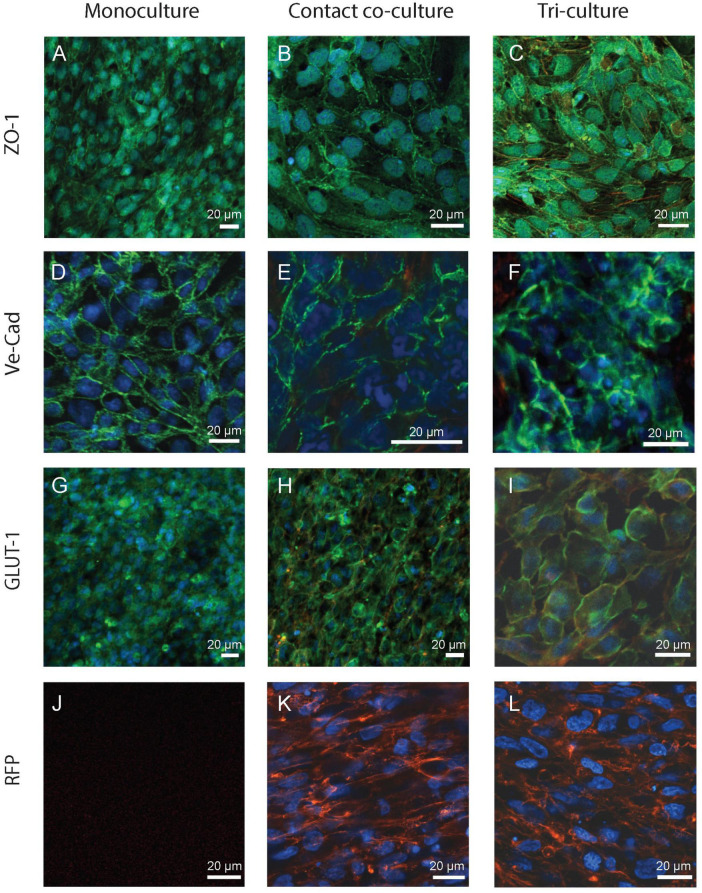
Fluorescence images of the *in vitro* BBB in the monoculture (left), co-culture (i.e., “contact” co-culture, center) and tri-culture configurations (right). Images were acquired from the insert porous membrane where endothelial cells and astrocytes were seeded and cultured as described in Section “Cell culture.” Cells were immunostained for key biomarkers representative of barrier integrity and glucose transport activity: **(A–C)** ZO-1, **(D–F)** VE-Cad, and transporters expression **(G–I)** GLUT-1. Cells were stained for each protein using AlexaFluor488 (green) and nuclei counterstained with Hoechst (blue). **(J–L)** Astrocytes were transfected with red fluorescent protein (RFP+), but they are absent in the monoculture condition **(J)**. Scale bar 20 μm.

Next, the monoculture, co-culture and tri-culture setups underwent optical characterization using confocal microscopy to visualize the expression of key biomarkers of the BBB. Despite the fact that the “indirect” contact co-culture exhibits higher (yet not statistically significant) TEER compared to the monoculture, there is almost no difference in permeability between the two setups. It is worth mentioning that within the human NVU a close physical interaction exists between the BBB and the surrounding pericytes/astrocytes (Iadecola, 2017; Bhalerao et al., 2020; Kadry et al., 2020) (i.e., end-foot in proximity of the endothelial cells); this scenario is not fully recapitulated by the “indirect” contact co-culture condition, where the Transwell membrane separates the apical endothelial monolayer from the astrocytes underneath. Thus, the “contact” co-culture setup (i.e., endothelial cells are seeded on top of astrocytes in inserts) was the only co-culture approach continued for further experiments and constituted the rationale for the development of the tri-culture model configuration. As shown in Figure 3, all setups showed a strong expression of relevant BBB markers involved in regulating barrier functional properties, specifically: TJs like ZO-1, which contribute to the formation of a highly selective and tight separation between the vasculature and brain compartments; adherens junctions like VE-cadherin, whose role is to ensure adhesion between endothelial cells and maintenance of the brain microenvironment; and finally influx/efflux transporters such as the glucose transporter GLUT-1, for glucose uptake. It is worth nothing that ZO-1 was expressed on the border of the cells, however, the staining across all the conditions also showed a cytoplasmic expression of this biomarker (Figures 3A–C), as observed elsewhere (Adriani et al., 2017; Biemans et al., 2017). In addition, VE-Cad expression pattern seemed to be uneven in the co-culture and tri-culture setups, yet this biomarker was found consistently expressed along the perimeter of the cells (Figures 3D–F).

### Neural cell viability and estimation of neurite outgrowth

**FIGURE 4:**
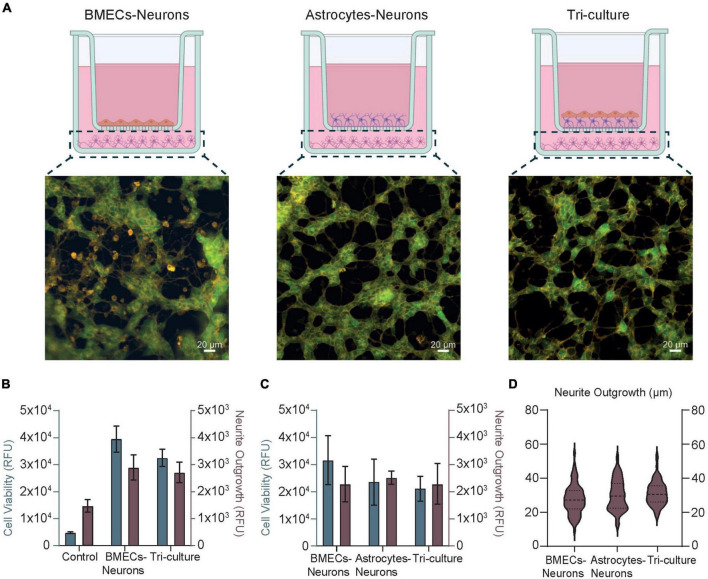
**(A)** Schematics and fluorescence images of SH-SY5Y cells representative of the neural compartment in the BMEC-neuron culture (left), astrocyte-neuron co-culture (center), and tri-culture (right) models at day 10 of cell culture. Created with www.biorender.com.. Cells were loaded with fluorescent dyes (Kaur et al., 2012) enabling a real-time visualization of neurons viability (green) and their neurites (orange) in the samples. **(B,C)** Bar Charts obtained upon fluorescence quantification of cell viability (gray) and neurite outgrowth (brown), both in arbitrary units (RFU). Control stands for cell-free samples loaded with the fluorescent dyes. **(D)** Box violin plots describing neurite outgrowth quantification (μm) obtained upon image analysis. Dash lines represent the median, and the dotted lines represent the 75 and 25% of the data distribution, respectively.

The selective and restrictive nature of the NVU toward foreign substances and pathogens is mostly dictated by the BMECs, which work in concert with other ancillary cells (e.g., pericytes, astrocytes, and neurons) in maintaining the brain homeostasis. Previous studies on advanced multicellular *in vitro* NVU models (Ito et al., 2019; Stone et al., 2019; Gericke et al., 2020) together with our findings reported above on TEER have demonstrated the effects of the cellular compartment interplay (i.e., vascular and neuronal) on barrier integrity. Compared to simplistic monoculture BBB models, sophisticated *in vitro* setups incorporating multiple NVU cell types seem to encourage or improve barrier properties, such as barrier integrity and resistance. However, very limited investigation has been carried out on the influence of a healthy BBB on neuronal behavior and phenotype. To this end, we developed a co-culture model of BMECs and neurons (i.e., without glia cells) and a co-culture model of astrocytes and neurons (i.e., without BBB) to be compared with the complete tri-culture NVU model (Figure 4A). Our aim was to evaluate any discrepancies in neural health and morphology among these conditions that can be inferred as BBB driven. By means of specific membrane and intracellular fluorescent dyes, which allow the simultaneous detection of viable neurons and their neurites in the sample (Kaur et al., 2012), we firstly assessed the direct influence of the BBB in the BMEC-neuron model on neuronal cell viability and neurite outgrowth. As reported in Figure 4B and Supplementary Figure 4, there is higher neural cell viability (level of green indicator dye) compared to the tri-culture setup, whereas the measured axonal lengths (relative fluorescence units, RFU) are comparable between samples. Similar results were obtained with the astrocyte-neuron model, in which the cell viability of the neurons was detected to be slightly lower than the BMEC-neuron setup yet closer to the tri-culture (Figure 4C). Interestingly, fluorescent images acquired for each cell model condition showed that neural cells of the tri-culture NVU model seem to possess a high number of neuronal processes and branched neurites (orange indicator dye) forming dense interconnected neural networks (Figure 4A and Supplementary Figure 4). All together, these findings may suggest that the presence of an endothelial barrier within the *in vitro* cell model strongly enhances neural health and viability, thus having beneficial influence on inducing neural networks formation and maturation. The decrease in the neural cell viability fluorescent signal found in both astrocytes-neurons and tri-culture may be due to the very high proliferation rate of the immortalized astrocytoma cell line used in our models, which likely took over the neuronal culture and partially affected the formation of a flat barrier monolayer in the tri-culture setup (see Section “Conclusion”). Finally, we further traced and estimated neurite projections in all the three cell model configurations to convert RFU in μm (see Section “Materials and methods” and Supplementary Figure 5). As reported in Figure 4D, we measured neurite length means of 27.53 ± 8.5 μm (range 9.248–54.642 μm) in the BMEC-neuron model, 30.14 ± 8.9 μm (12.4–52.4 μm) in the astrocyte-neuron co-culture and 31.17 ±6.9 μm (17.2–53.4 μm) in the tri-culture.

### Validation of the barrier integrity

**FIGURE 5:**
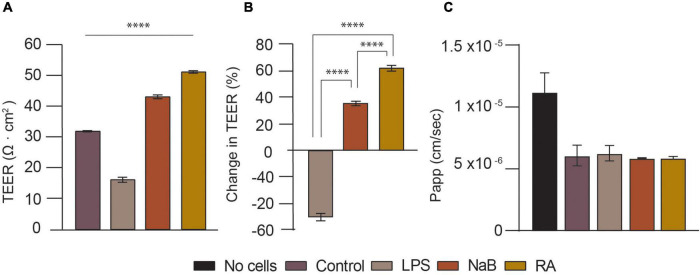
Validation of the tri-culture neurovascular unit (NVU) model by characterizing BBB permeability upon treatment with LPS, NaB, or RA for 24 h. **(A)** Transendothelial electrical resistance (TEER) measurements (Ω cm^2^) for NVU tri-culture setups used for BBB integrity evaluation: control (i.e., no treatment), LPS, NaB, and RA treated samples on day 14. The difference between the control and samples was analyzed using one-way ANOVA, with ^****^p < 0.0001 (Dunnett’s multiple comparison test). Error bars represent the standard deviation of three replicates for each condition (mean ±SD, *n* = 3). **(B)** Bar Chart showing the change in TEER (%) estimated for each treated tri-culture sample. The difference in % between treated samples was analyzed using one-way ANOVA, with ^****^p < 0.0001 (Tukey’s multiple comparison test). Error bars represent the standard deviation of three replicates for each condition (mean ±SD, *n* = 3). **(C)** Lucifer yellow (LY) flux and apparent permeability (Papp) bar charts for each tri-culture treated setup.

Blood-brain barrier (BBB) disruption or dysfunction generally correlates with lower TEER and greater permeability as ions can leak through the barrier tissue (Williams-Medina et al., 2021). Numerous substances, such as microbiota derived metabolites, soluble factors in the media or extracellular matrix (ECM), have been known to affect BBB integrity and thus permeability. For instance, 10 μM RA increased the TEER of iPSC-derived BBB models by more than 1,000 Ω cm^2^ (Butt et al., 1990; Sivandzade and Cucullo, 2018; Bhalerao et al., 2020). Conversely, BBB disruption occurs in response to pro-inflammatory stimuli, such as LPS and tumor necrosis factor–α (Lazear et al., 2015). Moreover, in recent years, the emerging area of gut-brain-microbiota research has revealed that gut bacteria can also influence BBB permeability by producing metabolites, such as Short Chain Fatty Acids (SCFAs) like NaB, which decreases BBB permeability and prevents BBB disruption after cerebral ischemia and LPS exposure (Braniste et al., 2014; Hoyles et al., 2018). Therefore, RA, LPS, and NaB were firstly chosen as soluble factors for validating the human NVU models developed herein by studying the effects of these soluble factors on the BBB integrity (Figures 5A,B).

In this experiment the tri-culture setups were used for barrier integrity assessment at day 14 of cell culture, following barrier steady-state which occurred from day 10 onward (i.e., TEER values reaching plateau). As illustrated in Figures 5A,B after 24 h of treatment with 10 μM RA, TEER increased by over 60% from 31.75 Ω cm^2^ (Control) to 51 Ω cm^2^ (^****^p < 0.0001). This is expected, as all-*trans*-retinoic acid (RA) is a metabolite of vitamin A and a well-known antioxidant, known to enhance the barrier properties of brain endothelial cells by regulating junctional proteins like occludin (Tóth et al., 2014). It was decided to treat the cells with 100 μM of NaB because that is close to the SCFAs potency range and human plasma concentration (Cleophas et al., 2019; Villabona-Rueda et al., 2019). After 24 h, this led to a 35% rise in TEER to 43 Ω cm^2^ (^****^p < 0.0001). This is not very different from a previous, similar study that also found that 24 h of treatment with 200 μM of NaB reduced the BBB permeability of brain endothelial cells in hanging inserts (Kim et al., 2021). It has been reported that butyrate restores the BBB, affects BBB permeability and has neuroprotective effects in murine models (Kim et al., 2021). On the contrary, LPS reduced TEER by almost 50% to just 16 Ω cm^2^ (^****^p < 0.0001). In another model, 24 h of LPS (100 ng/ml) treatment increased 7 kD FITC-Dextran permeability and reduced TEER by 15.85% (Ni et al., 2017), whereas this TEER reduction was between 30 and 50% in another study (Kim et al., 2021). As LPS did not cause morphologic changes in a previous study, its mechanism for increasing BBB permeability is not well understood. LPS, which can be found on gram-negative bacteria cell walls, is an endotoxin known to disrupt and penetrate the gut barrier in addition to impairing BBB integrity and being associated with brain diseases. Whilst there was no significant difference in LY permeability of the control compared to the cells treated with LPS, LPS seemed to cause a slight variability in LY Papp as seen in Figure 5C, whilst both RA and NaB treated samples exhibit a reduced Papp with respect to the control and the LPS condition.

## Conclusion

Representative, *in vitro* models of the NVU are crucial for improving our understanding of brain diseases and developmental phenomena, developing novel treatments, and testing the efficacy, toxicology and delivery of drugs. This paper details the development, optimization, characterization, and validation of a tri-culture NVU model, where human brain endothelial cells, astrocytes and neuronal cells are co-cultured on 24 well plates with hanging cell culture inserts. Monoculture, co-culture, and tri-culture configurations were compared, and TEER measurements showed that the tri-culture of hCMEC/D3, 1321N1 astrocytes and SH-SY5Y neurons gave the most optimal results with TEER values of almost 40 Ω cm^2^, which are in line with findings obtained from human immortalized cell lines reported in previous works. Results were verified using a LY permeability assay and immunostaining, which confirmed the expression of BBB markers and TJ formation. The model was then validated by treatment with various soluble factors with known effects on the BBB, and the model responded to this as predicted, with great sensitivity. We believe this *in vitro* biological model can be considered as a highly biomimetic but robust recapitulation of the human NVU aiming to unravel brain pathophysiology mechanisms as well as improve screening and delivery of therapeutics.

## Limitations and future work

Although we’ve demonstrated a highly usable and robust platform for studying the NVU, there is definitely room for improvement. Our recorded TEER measurements are still far from the human BBB *in vivo* reported measurements of TEER of over 1,000 Ω cm^2^ (Butt et al., 1990; Sivandzade and Cucullo, 2018; Bhalerao et al., 2020), and the expression of TJ proteins such as ZO-1 did not show high localization the cell periphery as might be expected. To further support and optimize our findings on BBB permeability as well as correlate them to our TEER results more accurately, future work will explore the use of functional assays for P-glycoprotein (p-GP). Specifically, rhodamine 123 dye would represent a complementary approach to perform transport studies on the p-GP activity and BBB efflux ratio in our model setups, similarly to previous studies (Tai et al., 2009; Simöes Da Gama and Morin-Brureau, 2022). One way of improving the model is by integrating more types of cells found in the NVU, such as pericytes and microglia, as well as refining cell culture conditions to extend the timeline for more endpoints to study. Despite several advantages associated with the use human immortalized cell lines for brain modeling (e.g., high proliferation rate), we did encounter some overgrowth issues of 1321N1 astrocytoma cells with respect to endothelial cells and neurons after 1 week of cell culture. Potential troubleshooting could be provided by replacing or combining immortalized cells with primary cell lines or iPSCs, both contributing to higher TEER values closer to the *in vivo* BBB. Furthermore, culturing the cells in their native 3D architecture to better mimic *in vivo* tissue physiology facilitates better intercellular signaling networks, cell-to-cell contact, and developmental processes (Moysidou et al., 2021; Barberio et al., 2022). 3D models composed of hydrogels or other biomaterials that mimic native biomechanical stiffness are more representative and have better BBB formation. The impact of substrate stiffness on the BBB has been reviewed in depth (Ferro et al., 2020). The flow of blood exerts shear stress of 10–20 dynes cm^–2^ on brain endothelial cells, and this has been recognized as a factor critical for inducing a mature BBB phenotype and regulating BBB integrity through TJ expression. Flow-based shear stress increased the TEER of hCMEC/D3 from less than 100 Ω cm^2^ to over 1,000 Ω cm^2^, so *in vitro* models can be vastly improved by introducing shear using dynamic, fluidic set-ups. *In vitro* NVU models can also be improved by automated, real-time monitoring of BBB properties and integrating electronic systems for monitoring neuronal function in health and disease aiming to generate highly representative and well characterized biological models. This could be achieved using 3D conducting scaffolds or hydrogels made of materials like carbon nanotubes or poly (3,4-ethylenedioxythiophene): poly (styrenesulfonate) (PEDOT:PSS), where the 3D substrate that the cells grow in is itself also a monitoring system for properties like cell growth and neuronal firing whilst providing a representative microenvironment for cells. Future work in our group will adapt our novel electronic transmembrane devices for NVU models such as those shown here (Pitsalidis et al., 2022).

## Data availability statement

The raw data supporting the conclusions of this article will be made available by the authors, without undue reservation.

## Author contributions

CB, AW, and YM conceived, executed, and analyzed results of experiments. RO conceived and directed research on the manuscript. CB, YM, AW, and RO wrote and edited the manuscript. All authors contributed to the article and approved the submitted version.
