# Supplementary material for: A human-derived neurovascular unit in vitro model to study the effects of cellular cross-talk and soluble factors on barrier integrity
Source: Front Cell Neurosci. 2022 Dec 1;16:1065193. doi: 10.3389/fncel.2022.1065193 (PMC9762047; doi:10.3389/fncel.2022.1065193)
Supplement: Supplementary file 1 [file Data_Sheet_1.docx]

# Supplementary Data

# Figure S1. Transendothelial electrical resistance (TEER) measurement

#

**Figure S1.** Mean TEER values (Ω·cm^2^) for hCMEC/D3 in monoculture, hCMEC/D3 and 1321N1 co-culture (hCMEC/D3+1321N1 as “contact” co-culture) and hCMEC/D3+1321N1/SHSY5Y tri-culture. The difference between monoculture, co- and tri-culture was assessed using a one-way ANOVA with **p<0.01, *p<0.05. The error bars represent the standard deviation of at least 3 replicates for each condition (mean ± SD).

**Figure S2. Transendothelial electrical resistance (TEER) measurement: static vs dynamic**

**Figure S2.** Mean TEER values (Ω·cm^2^) for hCMEC/D3 monoculture in static and dynamic conditions. The difference between the samples was evaluated using the unpaired t-test with **p<0.01 and *p<0.05.

**Figure S3. Lucifer Yellow (LY) permeability assay**

**Figure S3.** Lucifer Yellow (LY) flux and apparent permeability (P_app_) bar charts for monoculture, co-culture (including the hCMEC/1321N1) and tri-culture in static (left) and dynamic (right) condition. The error bars represent the standard deviation of at least 3 replicates for each condition (mean ± SD).

**Figure S4. Neural Health Assay**


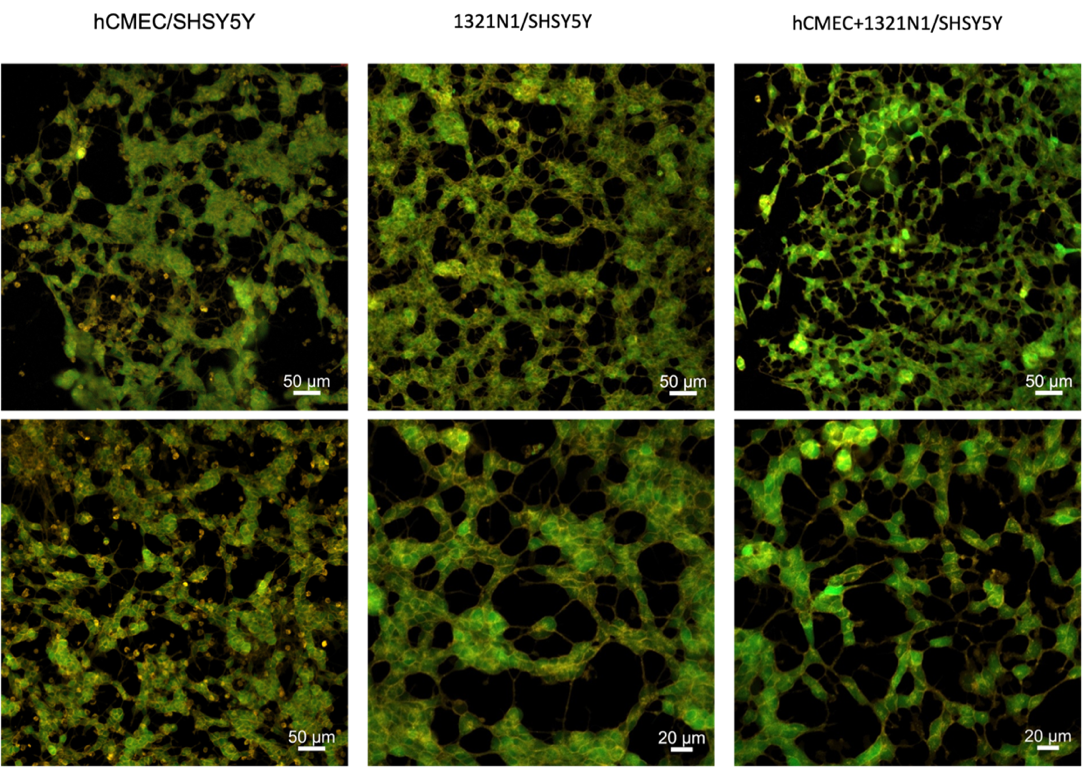


**Figure S4.** Confocal images of neural health assay for co-culture conditions BMECs-neurons(left), astrocytes-neurons (centre) and the tri-culture (right). Cell viability dye is shown in green; membrane stain dye (neurite outgrowth) is orange.

**Figure S5. NeuronJ tracings**


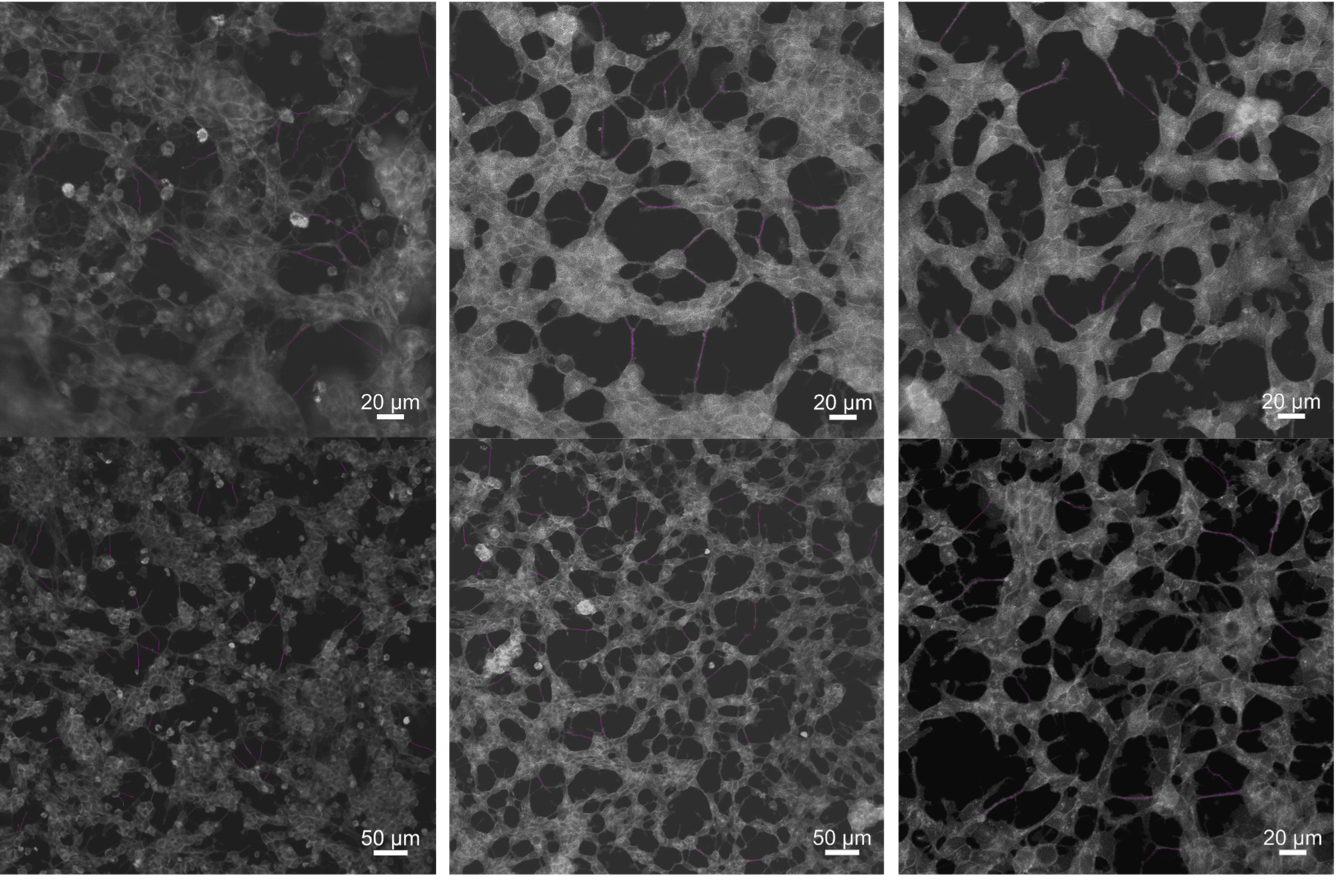


BMECs/neurons

Astrocytes/neurons

Tri-culture

**Figure S5.** Grayscale images obtained from neural health assay fluorescent images showing the semi-automated tracings (purple) used for neurite lengths measurement and quantification.
